# Revisiting Brain Atrophy and Its Relationship to Disability in Multiple Sclerosis

**DOI:** 10.1371/journal.pone.0037049

**Published:** 2012-05-15

**Authors:** Navid Shiee, Pierre-Louis Bazin, Kathleen M. Zackowski, Sheena K. Farrell, Daniel M. Harrison, Scott D. Newsome, John N. Ratchford, Brian S. Caffo, Peter A. Calabresi, Dzung L. Pham, Daniel S. Reich

**Affiliations:** 1 Department of Electrical and Computer Engineering, Johns Hopkins University, Baltimore, Maryland, United States of America; 2 Radiology and Imaging Sciences, National Institutes of Health, Bethesda, Maryland, United States of America; 3 Department of Neurophysics, Max Planck Institute for Human Cognitive and Brain Sciences, Leipzig, Germany; 4 Neuroradiology Division, Departments of Radiology and Radiological Science, Johns Hopkins University, Baltimore, Maryland, United States of America; 5 Department of Physical Medicine and Rehabilitation, Johns Hopkins University, Baltimore, Maryland, United States of America; 6 Department of Neurology, Johns Hopkins University, Baltimore, Maryland, United States of America; 7 Department of Biostatistics, Bloomberg School of Public Health, Johns Hopkins University, Baltimore, Maryland, United States of America; 8 Center for Neuroscience and Regenerative Medicine, The Henry M. Jackson Foundation for the Advancement of Military Medicine, Bethesda, Maryland, United States of America; 9 Neuroimmunology Branch, National Institute of Neurological Disorders and Stroke, National Institutes of Health, Bethesda, Maryland, United States of America; Innsbruck Medical University, Austria

## Abstract

**Background:**

Brain atrophy is a well-accepted imaging biomarker of multiple sclerosis (MS) that partially correlates with both physical disability and cognitive impairment.

**Methodology/Principal Findings:**

Based on MRI scans of 60 MS cases and 37 healthy volunteers, we measured the volumes of white matter (WM) lesions, cortical gray matter (GM), cerebral WM, caudate nucleus, putamen, thalamus, ventricles, and brainstem using a validated and completely automated segmentation method. We correlated these volumes with the Expanded Disability Status Scale (EDSS), MS Severity Scale (MSSS), MS Functional Composite (MSFC), and quantitative measures of ankle strength and toe sensation. Normalized volumes of both cortical and subcortical GM structures were abnormally low in the MS group, whereas no abnormality was found in the volume of the cerebral WM. High physical disability was associated with low cerebral WM, thalamus, and brainstem volumes (partial correlation coefficients ∼0.3–0.4) but not with low cortical GM volume. Thalamus volumes were inversely correlated with lesion load (*r* = −0.36, *p*<0.005).

**Conclusion:**

The GM is atrophic in MS. Although lower WM volume is associated with greater disability, as might be expected, WM volume was on average in the normal range. This paradoxical result might be explained by the presence of coexisting pathological processes, such as tissue damage and repair, that cause both atrophy and hypertrophy and that underlie the observed disability.

## Introduction

Multiple sclerosis (MS) causes multifocal white matter (WM) lesions that are easily visible on MRI and that are characterized by a varied array of pathologic processes, including inflammation, demyelination, remyelination, edema, axonal damage, and gliosis [1]. All of these processes can cause substantial changes in tissue volume. Multifocal gray matter (GM) lesions are present as well, but they are less conspicuous on MRI due to smaller increases in water content; indeed, on conventional MRI, GM involvement is mostly characterized by volume loss [2]–[4] that begins early in the disease course [5], [6]. GM volume loss includes both cortical thinning [7], [8] and subcortical atrophy [9]–[12]. A central open question is the extent to which WM and GM damage interact with one another and contribute to disability in MS [13].

Disability in MS is commonly assessed via the Expanded Disability Status Scale (EDSS) and the MS Functional Composite (MSFC) [14]; the MS Severity Score (MSSS) [15] is another useful measure. The relationships between each of these measures and volumes of lesions and/or individual brain structures have been studied previously. For example, there is an inverse correlation between EDSS and normalized measures of brain volume [6], [16] as well as volumes of WM and GM, both globally [2], [17], [18] and regionally [8], [19]. However, associations between volumetric abnormalities and measures of disability in individual limbs, such as ankle dorsiflexion strength and great-toe vibration thresholds, have been poorly characterized if at all.

Missing from the literature is a cross-sectional characterization of brain structure volumes and their association with a wide range of disability scores in a single representative cohort. Specifically, studies of subcortical GM structures have been limited either to a few structures at a time [9]–[11] or to a few disability scores [12]. Moreover, the relationship of brainstem atrophy to disability has not been completely characterized [12], [20], [21]. This is important because in MS the spinal cord is an important substrate of disability, and brainstem and spinal cord volumes are correlated [21].

To address this gap in the literature, the current study investigates brain structure volumes (including cortical and subcortical GM structures, cerebral WM, CSF, and brainstem) and volumes of WM lesions, as well as the relationship between brain volumetric abnormalities and strength, cognitive, and sensory impairment measures. The study group is a representative sample of MS cases (including all three major MS subtypes) at a tertiary referral center. The cases represent a wide spectrum of disability, age, and disease duration. Results from MS cases are compared with those from a matched group of healthy volunteers. Scans were analyzed using Lesion-TOADS, our previously described, freely available software package that segments the brain into its major structures while simultaneously delineating WM lesions [22]. The major advantage of Lesion-TOADS is that it provides a comprehensive account of brain structure volumes with no manual interaction, making it particularly useful for large-scale studies.

## Materials and Methods

### Ethics Statement

Protocols were approved by the Institutional Review Boards at the Johns Hopkins University School of Medicine and the F.M. Kirby Research Center for Functional Brain Imaging at the Kennedy Krieger Institute. Written, informed consent was obtained from all participants.

### Participants

The study group consisted of 37 healthy volunteers (22 women) with a mean age of 37 years (range: 23–63) and 60 MS cases (45 women) with a mean age of 43 years (range: 20–68) and mean disease duration of 8 years (range: 0–30). There were 43 cases of relapsing remitting (RRMS), 9 cases of secondary progressive MS (SPMS), and 8 cases of primary progressive MS (PPMS). MS cases were referred by treating neurologists in the Johns Hopkins MS Center. We did not enroll prospective participants who had additional neurological diagnoses that could cause abnormalities on brain MRI. 53 MS cases were taking disease-modifying therapy at the time of the study; the others were untreated at the time of scanning. We collected clinical data at the Johns Hopkins University and MRI scans at the F. M. Kirby Research Center for Functional Brain Imaging at the affiliated Kennedy Krieger Institute.

### Impairment measures

We performed the MSFC and EDSS within 30 days of the MRI scans on all the MS cases. We used the protocol prescribed in the MSFC Administration and Scoring Manual [23] to administer the individual MSFC components (9-hole peg test, PASAT-3, and timed 25-foot walk) and to calculate the final summary *z*-scores. For all statistical analyses, we averaged the 9-hole peg-test times across the dominant and non-dominant hands. We also computed the MSSS score, which relates scores on the EDSS to the distribution of disability in patients with comparable disease durations [15]. We assessed vibration sensation and ankle dorsiflexion strength within a week of the MRI scans to quantify sensorimotor dysfunction in a subset of 56 subjects from the MS cohort (41 RRMS, 8 SPMS, 7 PPMS). We quantified vibration sensation thresholds for both great toes using the Vibratron II device (Physitemp, Huron, NJ) [24]. For this test, each subject was evaluated using a two-alternative forced choice procedure [24]. We quantified ankle dorsiflexion strength using a Microfet2 hand-held dynamometer (Hoggan Health Industries, West Jordan, UT) [25]. For this test, the average of two maximal efforts for each ankle was calculated and recorded. For subsequent analysis, we selected the value obtained from the side with the poorer sensory or motor score [26]. Table 1 shows the impairment statistics in the MS cohort. We did not test for impairments in the healthy volunteers.

**Table 1:** Cohort demographic data.

Impairment measure	All	RRMS	SPMS	PPMS
**EDSS**	2.7 (0–6.5)^a^	2.0 (0–6.0)^a^	6.0 (2.5–6.0)^a^	3.7 (2.5–6.5)^a^
**MSSS**	4.2 (0.1–9.1)^a^	3.7 (0.1–8.6)^a^	5.4 (2.0–6.5)^a^	6.9 (2.7–9.1)^a^
**MSFC**	0 (0.7)^b^	0.1 (0.7)^b^	−0.3 (0.4)^b^	−0.4 (0.7)^b^
**25-foot walk test (** ***seconds*** **)**	4.6 (4.0–5.5)^c^	4.4 (3.9–4.9)^c^	5.9 (5.3–6.2)^c^	7.1 (4.4–10.2)^c^
**9-hole peg test (** ***seconds*** **)**	21.0 (19.4–24.1)^c^	20.0 (19.2–22.3)^c^	22.3 (20.1–29.2)^c^	24.8 (21.2–25.5)^c^
**PASAT-3, ** ***max = 60***	50 (43–57)^c^	51 (41–56)^c^	46 (43–56)^c^	52 (47–60)^c^
**vibration sensation** *	4.4 (1.5–16.4)^c^	2.2 (1.3–5.6)^c^	9.8 (6.0–21.0)^c^	18.4 (15.5–44.0)^c^
**ankle strength measure (** ***pounds*** **)**	42.2 (35.1–52.7)^c^	44.5 (40.0–54.0)^c^	35.5 (28.2–44.4)^c^	32.5 (26.0–42.2)^c^

a *median (range),*

b *mean z-score (standard deviation),*

c *median (interquartile range).*

* Vibration units are amplitudes, proportional to the square of the applied voltage.

*Abbreviations*. *RRMS*, relapsing remitting multiple sclerosis. *SPMS*, secondary progressive multiple sclerosis. *PPMS*, primary progressive multiple sclerosis. *CIS*, clinically isolated syndrome. *EDSS*, Expanded Disability Status Scale. *MSSS*, Multiple Sclerosis Severity Score. *MSFC*, Multiple Sclerosis Functional Composite. *PASAT-3*, Paced Auditory Serial-Addition Task, 3 second version.

### MRI protocol and image analysis

We acquired all images on a 3 tesla scanner (Intera, Philips Medical Systems). We used 2 axial, whole-brain sequences without gaps for this study: multislice, T2-weighted fluid-attenuated inversion recovery (FLAIR; acquired resolution: 0.8×0.8×2.2 or 0.8×0.8×4.4 mm; TE: 68 ms; TR: 11 s; TI: 2.8 s; SENSE factor: 2; averages: 1); and 3D magnetization-prepared rapid acquisition of gradient echoes (MPRAGE; acquired resolution: 1.1×1.1×1.1 mm; TE: 6 ms; TR: ∼10 ms; TI: 835 ms; flip angle: 8 deg; SENSE factor: 2; averages: 1).

**Figure 1:**
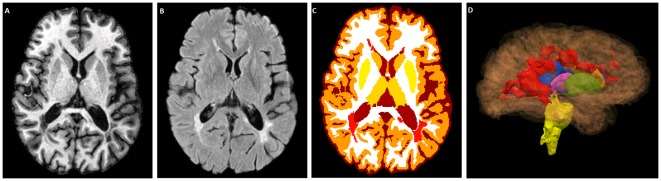
Lesion-TOADS segmentation results. Representative slices from the T1-weighted (A) and FLAIR (B) scans of one of the MS cases. Lesions are depicted in red (C). 3D rendering of ventricles (blue), putamen (green), caudate (orange), thalamus (pink), brainstem (yellow), and lesions (red), generated by Lesion-TOADS (D).

We analyzed the images with the TOADS-CRUISE software package (http://www.nitrc.org/projects/toads-cruise). Our previously published and validated segmentation method, Lesion-TOADS [22], segmented the brain into its component structures while simultaneously delineating the lesions. Lesion-TOADS combines the intensity information from MPRAGE and FLAIR images with statistical and topological atlases to yield the locations and volumes of FLAIR-hyperintense WM lesions, sulcal CSF, ventricular CSF, cortical GM, total cerebral WM (including lesions), cerebellar GM, cerebellar WM, putamen, caudate, thalamus, and brainstem (Fig. 1). Before applying Lesion-TOADS, we first removed the skull and extracranial tissues, again using an automated method [27] based on the MPRAGE scan and then applied the resulting brain-and-CSF mask to the coregistered FLAIR scan. Next, we rigidly registered the stripped MPRAGE scans to a single image atlas (“JHU_MNI_SS_T1”) that is available for download (http://www.mristudio.org). This atlas, which contains 1 mm isotropic voxels in a field-of-view of 181×217×181 mm, has been registered to the standard Montreal Neurological Institute-152 atlas. The FLAIR images were also transformed to this space. As the imaging field of view affects the measurement of brainstem volume, we cropped all the images on a certain location in the MNI space in the inferior part of the brain (z = 25). After manual review of the segmentation results by an experienced observer (DSR), we identified 12 MS cases with low lesion load whose lesion volumes were overestimated by the Lesion-TOADS procedure when run with the default settings. In these cases, we modified the default settings by decreasing the parameter controlling the intensity variance of lesions, which resulted in successful segmentation. Only four cases required manual editing of the lesion masks.

After segmenting brain structures and lesions, we computed the cerebral volume fraction (CVF) by normalizing the sum of the volumes of all brain structures except cerebellum to the intracranial volume. The CVF is analogous to the brain parenchymal fraction, which was previously defined based on a different segmentation technique [28]. We also normalized individual brain structure volumes in the same fashion.

One of the sources of inconsistent results in previous studies is different segmentation methods [17]. We therefore performed a separate analysis of our data using FSL's software tools (http://fmrib.oxo.ax.uk/fsl), which are commonly used to measure brain volumes in MS [28], [29]. We could not directly use SIENAX [30], FSL's tool for cross-sectional analysis of brain volumes in the brain, since its skull-stripping step performed poorly on our data. We therefore used the skull-stripping tool in the TOADS-CRUISE package to extract the brain, as described above, and provided the results to FSL's FAST algorithm [31] (the same segmentation tool used in SIENAX) for tissue classification. We also used the lesion masks generated by Lesion-TOADS to remove the lesions prior to final segmentation, as lesions can result in misclassifications within FAST; SIENAX uses the same approach to handle the lesions. The only major difference between our approach for using FSL's FAST for brain tissue classification and SIENAX is that SIENAX performs normalization to a common space.

### Statistical analysis

We performed the statistical analysis in R (version 2.14.1). Except for lesion volume, for which we applied a log transform, each of the structure volume distributions was not significantly different from Gaussian (Lilliefors test). We therefore assessed the significance of differences in structure volumes between the MS and healthy-volunteer groups with multivariable linear regression models accounting for sex and age. We also performed a partial correlation analysis to investigate the associations between impairment measures and structure volumes. To study the effect of sex on the interaction between disability and atrophy, we ran a regression model with an interaction term between sex and structure volumes.

We used linear regression to investigate the effect of the two different FLAIR slice thicknesses on the structure and lesion volumes; we did not find any significant association, so FLAIR resolution was not included as a covariate in our analysis. Because this was an exploratory study applying a new segmentation method, and because brain structure volumes tend to be correlated with one another (see Table 2), we did not apply a strict correction for multiple comparisons and instead set the threshold significance level to á = 0.01. Instead, we carefully account for all tests performed and report p-values so that readers can calibrate significance relative to Bonferroni corrections.

**Table 2:** Partial correlations of normalized brain structure with one another in MS cases.

	white matter	caudate	putamen	thalamus	brainstem	ventricles	white matter lesions
**cortical gray matter**	0.16 (0.23)	**0.44 (<0.001)**	**0.38 (0.002)**	**0.37 (0.003)**	0.18 (0.18)	−0.29 (0.02)	0.13 (0.35)
**white matter**		**0.38 (0.002)**	0.03 (0.83)	**0.60** (**<0.001**)	**0.36 (0.004)**	**−0.67 (<0.001)**	−0.27 (0.04)
**caudate**			**0.46 (<0.001)**	**0.52 (<0.001)**	**0.34 (0.006)**	**−0.61 (<0.001)**	**−0.32 (0.01)**
**putamen**				**0.43 (<0.001)**	0.24 (0.06)	**−0.33 (0.009)**	−0.07 (0.62)
**thalamus**					**0.57 (<0.001)**	**−0.72 (<0.001)**	**−0.36 (0.005)**
**brainstem**						**−0.44 (<0.001)**	−0.21 (0.12)
**ventricles**							**0.40 (<0.001)**
**cerebral volume fraction**							**−0.45 (<0.001)**

Results are adjusted for linear effects of age and sex. The *p*-values are shown in parentheses, and results with *p*<0.01 are shown in boldface.

## Results

### Brain volume analysis

**Figure 2:**
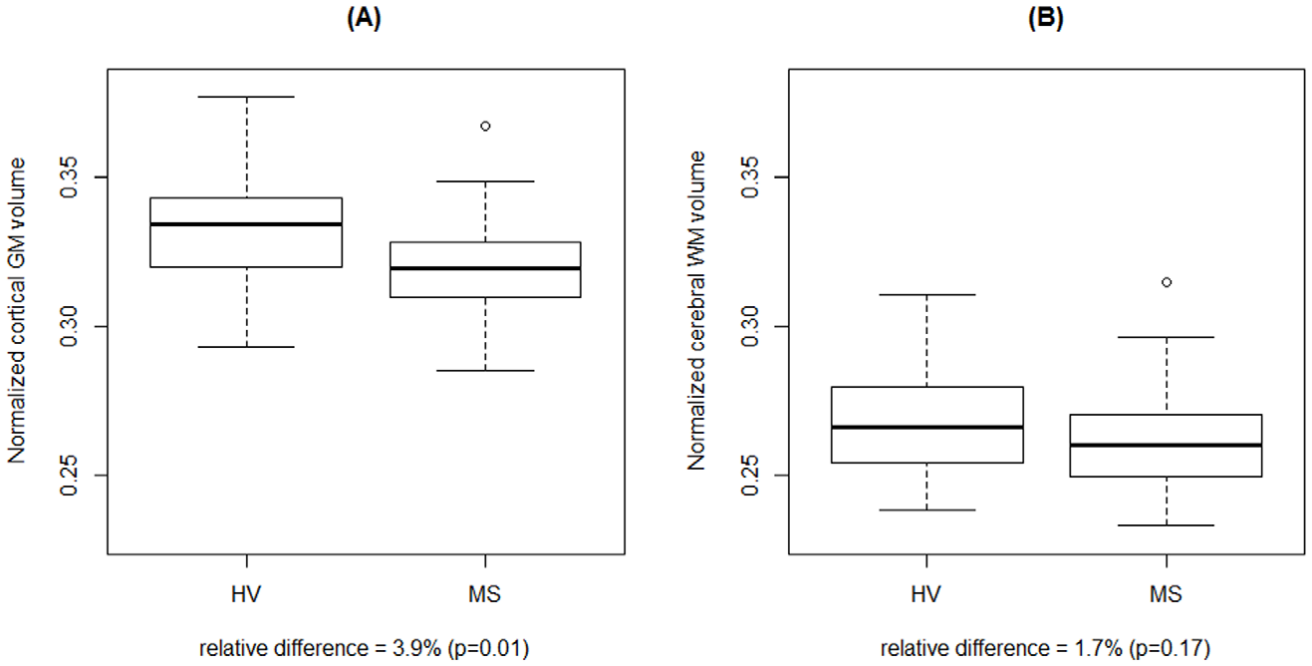
Distributions of normalized cortical gray matter (A) and cerebral white matter (B) volumes in healthy volunteers vs. MS cases. White matter volume is not significantly atrophic in the MS group, whereas cortical GM volume is substantially lower in MS.

After additive adjustments for sex and age through a linear model, all cortical and subcortical GM structure volumes, as well as CVF, were significantly smaller in the MS cohort (Table 3). Brainstem volume trended toward smaller values in MS, whereas ventricle volume trended towards larger values in MS; in fact, the latter had the largest relative volume change (38% larger in MS). Total cerebral WM volume, which includes lesion volume as well as extralesional or “normal-appearing” white matter, was not significantly reduced in MS (Fig. 2).

**Table 3:** Normalized brain structure volumes.

	HV mean (SD)	MS mean (SD)	difference	p-value
**cortical gray matter**	0.3324(0.0179)	0.3193 (0.0157)	−3.9%	**0.01**
**white matter**	0.2670 (0.0175)	0.2625 (0.0169)	−1.7%	0.17
**caudate**	0.0051 (0.0007)	0.0042 (0.0006)	−18.8%	**<0.001**
**putamen**	0.0064 (0.0006)	0.0059 (0.0006)	−8.7%	**<0.001**
**thalamus**	0.0082 (0.0007)	0.0073 (0.0009)	−10.8%	**<0.001**
**brainstem**	0.0129 (0.0012)	0.0122 (0.0011)	−5.4%	0.02
**ventricles**	0.0123 (0.0065)	0.0169 (0.0072)	37.7%	0.02
**cerebral volume fraction**	0.7536 (0.0209)	0.7368 (0.0263)	−2.2%	**0.006**

Results with *p*<0.01 are shown in bold face. *Abbreviations*. *HV*, healthy volunteers. *MS*, multiple sclerosis. *SD*, standard deviation.

### Correlation analysis

Lesion volume correlated most strongly with ventricular volume and CVF but also with caudate and thalamus volumes (Table 2). Table 4 shows the correlation between each of the impairment measures and lesion volume, structure volumes, and CVF, all adjusted for the effects of age and sex. EDSS, MSFC, and 9-hole peg test time correlated with volumes of WM. 9-hole peg test time and MSFC also correlated with CVF, ventricle, and thalamus volumes. Both EDSS and 25-foot walk time correlated with brainstem volume. 25-foot walk time also correlated with thalamus volume. Lesion volume only correlated with 9-hole peg test time. Among the targeted sensorimotor measurements, ankle dorsiflexion strength correlated with thalamus volume and CVF. There were no correlations between brain structure volumes and great toe sensation threshold or PASAT-3 score.

**Table 4:** Partial correlations of impairment measures with normalized structure volumes.

	cortical gray matter	white matter	caudate	putamen	thalamus	brainstem	ventricles	cerebral volume fraction	white matter lesions
**toe sensation**	0.08 (0.59)	−0.10 (0.46)	−0.05 (0.74)	−0.18 (0.20)	−0.16 (0.25)	−0.24 (0.07)	−0.01 (0.97)	−0.08 (0.55)	0.19 (0.17)
**ankle dorsiflexion**	0.09 (0.54)	0.20 (0.14)	0.14 (0.32)	0.01 (0.92)	**0.44 (<0.001)**	0.24 (0.07)	−0.29 (0.03)	**0.33 (0.01)**	−0.25 (0.07)
**EDSS**	0.11 (0.40)	**−0.40 (0.001)**	−0.02 (0.86)	0.17 (0.20)	−0.16 (0.22)	**−0.34 (0.007)**	0.24 (0.06)	−0.27 (0.04)	0.06 (0.68)
**MSSS**	−0.10 (0.44)	−0.23 (0.07)	−0.06 (0.65)	0.05 (0.72)	−0.08 (0.55)	−0.30 (0.02)	0.16 (0.23)	−0.22 (0.09)	0.07 (0.65)
**9-hole peg test time**	0.00 (0.97)	**−0.45 (<0.001)**	−0.20 (0.12)	0.01 (0.97)	**−0.35 (0.005)**	−0.27 (0.03)	**0.47 (<0.001)**	**−0.46 (<0.001)**	**0.34 (0.008)**
**25-foot walk time**	−0.01 (0.95)	−0.23 (0.08)	−0.03 (0.84)	−0.04 (0.75)	**−0.32 (0.01)**	**−0.32 (0.01)**	0.23 (0.07)	−0.22 (0.10)	0.06 (0.67)
**PASAT-3**	0.07 (0.61)	0.10 (0.45)	−0.04 (0.75)	0.09 (0.48)	0.11 (0.42)	0.02 (0.88)	−0.21 (0.11)	0.17 (0.21)	0.15 (0.28)
**MSFC**	0.05 (0.70)	**0.35 (0.005)**	0.07 (0.59)	0.05 (0.69)	**0.34 (0.007)**	0.25 (0.05)	**−0.42 (0.001)**	**0.39 (0.001)**	−0.11 (0.41)

Results are adjusted for linear effects of age and sex. The *p*-values are shown in parentheses, and results with *p*<0.01 are shown in boldface. *Abbreviations*. *EDSS*, Expanded Disability Status Scale. *MSSS*, MS Severity Score. *MSFC*, Multiple Sclerosis Functional Composite *z*-score. *PASAT-3*, Paced Auditory Serial Addition Test, 3-second version.

In the MS cohort, men scored worse than women on the tests involving the assessment of physical disability except for ankle dorsiflexion, where men were stronger; however, only the group differences for MSSS and ankle dorsiflexion strength were close to the 0.05 significance level (39% higher MSSS in men; *p* = 0.05 and 30% higher ankle dorsiflexion strength in men; *p* = 0.05). We therefore further investigated the effect of sex by examining its interaction with structure volumes. In this analysis, we found a significant interaction effect in the association of ankle dorsiflexion strength with cerebral WM volume (*p* = 0.005) and toe sensation threshold with ventricles volume (*p* = 0.01).

### Comparison with an alternative analysis method

We performed a complementary analysis of our data with FSL's commonly employed software tools to measure brain volumes. In this analysis, we used Lesion-TOADS to isolate and remove the influence of WM lesions on the volume measurements. The FSL-derived brain-volume results were similar to those obtained with Lesion-TOADS, showing significant atrophy in GM (6.30% lower in MS; *p*<0.001) but not WM (1.09% higher in MS; *p* = 0.14). However, using FSL's FAST, none of the impairment measures in the MS cohort associated with WM or GM volume (Table 5).

## Discussion

Brain atrophy is a common feature in MS, a disease that is otherwise spatially and temporally heterogeneous. We undertook this work to evaluate brain structure and lesion volumes in a representative MS cohort at an academic medical center and to assess the relationships between volumetric abnormalities and clinical disability. One novel aspect of our study is that we consider simultaneously a spectrum of disability measures and a wide palette of brain structures. This analysis allows the findings of disparate studies on distinct cohorts to be tested in a single group of MS cases and healthy volunteers. A surprising result of our study is the finding that the MS cohort had normal WM volume on average, but that individuals with lower WM volume had more physical disability. We consider this apparent paradox later in the Discussion.

### Brain atrophy

We found significantly reduced volumes of both cortical and subcortical GM structures (including thalamus, caudate, and putamen) in MS cases. Our results also suggest that MS cases have lower brainstem volume and higher ventricular volume (*p* = 0.02). We did not find a significant change of the cerebral WM volume in MS. As previously reported [12], [32], [33], ventricular volume was abnormally high, probably on an ex vacuo basis. As a result, we found a significant reduction in the cerebral volume fraction (the ratio of brain parenchymal volume to intracranial capacity), a measure that is routinely used as a single-time-point estimate of brain atrophy in cross-sectional MS studies and that is considered a reasonable marker of neurodegeneration [2], [3], [5], [16], [17], [34], 35.

Whether or not MS is characterized by specific WM atrophy has been a matter of some debate [13]. Although we did not find evidence for overall WM volume loss in our cohort, we did find, surprisingly, that MS patients with less WM volume tend to have more disability. In GM, on the other hand, we found evidence of substantial volume loss, extending previous results by focusing on cortical volume rather than thickness [7], [8], [12]. (Though volume and thickness are related quantities, thinning without volume reduction might occur, for example, in the presence of a more complex cortical folding pattern. However, we did not find this to be the case in our cohort.)

Most prior studies of subcortical GM volumes in MS (with one notable exception; see [12]) have been limited to individual structures. In our study, we found that all subcortical GM structures were significantly atrophic in MS. Previous studies have identified that the thalamus is especially damaged in MS, probably reflecting its central role at the nexus of brain information processing. Caudate nucleus atrophy has been found inconsistently [12], but the 19% reduction we found here is similar to the extent of caudate atrophy reported in a previous study [10]. We also found a 9% reduction in putamen volume, confirming previous results [12].

In evaluating atrophy within and between groups, one should always consider the effect of variability in the signal intensity of individual structures, especially at their borders. This is always a challenge in performing segmentation, regardless of whether the segmentation procedure is fully automated, semi-automated, or manual. In addition to variability within the population, there may be group-level effects due to signal intensity differences, which might lead to consistently different segmentation results in the MS and healthy volunteer groups. The thalamus and its interface with the internal capsule present particular problems for segmentation routines. This is because signal intensity on T1-weighted images in the thalamus can be quite high, probably due to a relatively high myelin content. In MS, demyelination in the thalamus would reduce its signal intensity on T1-weighted images, leading to a more accurate delineation of the thalamus from the adjacent internal capsule and consequently artifactually higher thalamic volumes (and lower white matter volumes) compared to healthy volunteers. Thus, it is possible that thalamic volumes are actually lower in MS, and white matter volumes higher (and thus even less different from healthy volunteers), than we report here.

### Association between lesion load and brain structure volumes

WM lesion load correlated most strongly with ventricle volume (*r* = 0.40, *p*<0.001) and CVF (*r* = −0.45, *p*<0.001), confirming previous findings [5], [17], [36]–[38]. Among the GM structures we studied, caudate and thalamus volumes were correlated with lesion load. The association between thalamic atrophy and lesion load in MS is consistent with prior results and might be expected from the widespread connections between the thalamus and many areas of WM, where the lesions we detected are located [11]. In our study, although the association between lesion load and WM volume did not reach the 0.01 significance level that we set, it showed a trend toward a negative correlation between the two (*r* = −0.27, *p* = 0.04). Most previous studies did not find the inverse correlation between total WM volume and lesion load that we observed [3], [5], [39], [40]. This finding can be explained by the fact that MS lesions cause local tissue destruction (demyelination and axonal loss).

### Volumetric correlates of impairment measures

The most unexpected finding in our cohort was an inverse correlation between physical disability (EDSS and 9-hole peg test time) and WM volume in the absence of overt WM atrophy on the whole-group level. This apparent paradox has several possible explanations. The simplest possibility is that subtle WM atrophy was in fact present in our cohort but in too limited an extent to have been detected in our study. The trending inverse correlation between WM volume and lesion load provides some evidence for this possibility because it implies that individuals with high lesion loads are likely to have more WM atrophy. However, this explanation begs the question of why WM atrophy is so limited even in the presence of a high lesion load, a question we address in the next paragraph. A second explanation for the paradox is that individuals with lower premorbid WM volume are more likely to suffer disability, though this seems implausible. Finally, from a technical point of view, it is also possible that although we included age as a linear covariate in our regression models, subtle age differences not properly accounted for in the models might have obscured a difference in WM volume. Consistent with this possibility are the findings of Bartzokis et al. [41] that WM volume slightly increases between the second and fifth decades before starting to decrease, suggesting that linear modeling may be limited.

**Table 5:** Partial correlations of impairment measures with normalized structure volumes computed by FSL FAST segmentation tool.

	gray matter	white matter
**toe sensation**	−0.08 (0.55)	0.01 (0.92)
**ankle dorsiflexion**	0.21 (0.11)	0.00 (0.96)
**EDSS**	−0.15 (0.27)	−0.08 (0.53)
**MSSS**	−0.25 (0.05)	−0.08 (0.56)
**9-hole peg test time**	−0.26 (0.05)	−0.14 (0.29)
**25-foot walk time**	−0.16 (0.24)	−0.10 (0.43)
**PASAT-3**	0.06 (0.67)	−0.02 (0.89)
**MSFC ** ***z*** **-score**	0.23(0.08)	0.10 (0.45)

Results are adjusted for linear effects of age and sex. The *p*-values are shown in parentheses, and results with *p*<0.01 are shown in boldface. *Abbreviations*. *EDSS*, Expanded Disability Status Scale. *MSSS*, MS Severity Score. *MSFC*, Multiple Sclerosis Functional Composite. *PASAT-3*, Paced Auditory Serial Addition Test, 3-second version.

However, there is a more interesting and compelling explanation for the dissociation between WM atrophy (absent) and significant WM correlation with disability (present). If WM damaged by MS lesions undergoes a process that is associated with tissue hypertrophy (such as remyelination and gliosis), the effects on WM volume of tissue damage such as axonal loss and demyelination might be offset. If the hypertrophy is primarily associated with tissue repair, so that people with less disability have a more efficient repair process, the end result would be a near preservation of total WM volume in those individuals despite a cohort-wide correlation with disability scores – exactly the situation we encountered. In other words, the combination of MS-related tissue damage and repair would essentially induce a resorting of WM volumes across the population without overt atrophy. The residual inverse correlation between WM volume and lesion load would in this scenario reflect failure of the repair process in some individuals.

Also surprising to us was the absence, in our cohort, of a strong association between GM volume (either total, cortical, or subcortical) and EDSS; this finding contrasts with some previous results [2], [17], [18], [42], though not with all [43]. In addition to EDSS, the 25-foot walk and 9-hole peg test times have previously been reported to be associated with GM but not WM volume in RRMS [17], [18]. The lack of association between cortical GM atrophy and impairment measures in our data might be due to the fact that overall evaluation of cortical GM volume, particularly with respect to the cortex, blurs regional associations with performance on each of the examined tasks. If this is the case, a more detailed analysis of cortical regions would reveal some associations.

Despite the lack of association between cortical GM and disability in our study, we found out that thalamus volume is inversely associated with ankle dorsiflexion strength, 9-hole peg test time, and 25-foot walk time. Thalamic atrophy has been widely studied in MS, most commonly in the context of cognitive impairment [11], [44]. Our results point to a more complex effect of thalamic atrophy in MS, specifically in the context of motor disability. This fits with published reports that although thalamus is not involved in generating motor function, it is highly involved in motor control [45].

Similar to the report in [19], our study also revealed that physical disability (including EDSS and 25-foot walk time) is correlated with reduced brainstem volume. MSSS and 9-hole peg test time also showed borderline associations with brainstem atrophy. These associations can be understood in terms of the major role played by the brainstem, a mixed GM and WM structure containing many long tracts that travel in close proximity to one another, in physical functioning. Our results suggest that brainstem atrophy might be a candidate marker of physical disability in MS and are concordant with findings from another report that studied volume loss in the medulla oblongata [21]. As suggested by the authors of that study, some of the spinal-cord damage that is strongly associated with physical disability might be captured in measures of brainstem volume.

Despite its association with motor dysfunction, brainstem volume was not significantly correlated with sensory abnormalities (*r* = −0.24, *p* = 0.07). This is surprising because the WM fibers that mediate vibration sensation are located in the dorsal columns of the spinal cord and pass through the brainstem en route to the thalamus [26]. Sensory abnormalities were also not associated with supratentorial brain volumes, including the thalamus.

Due to time limitations, our evaluation of cognitive dysfunction in MS was unfortunately limited to the PASAT-3, and we did not observe correlations between scores on that test and any of our volumetric measurements. This includes the thalamus, the volume of which has been correlated with PASAT-3 scores in other studies [11], [44]. It is important to mention here that the cohorts investigated in those studies had substantially lower PASAT scores (35±14 in [11] and 42±16 in [44]) than our cohort (49±9). Noting that the amount of thalamic atrophy reported by Houtchens et al. is higher than what we reported here (17% vs. 11%), one might conclude that such an association exists when the cognitive impairment is more severe. However, a more thorough investigation of the relationship between brain atrophy and cognitive disability in MS is necessary to confirm this hypothesis.

Finally, we investigated whether sex plays an important role in mediating the correlations between brain volumes and impairment by including interaction terms in our regression models. The analysis uncovered significant interaction effects in a few cases, but not across the board. Some of these interactions may be explained by unbalanced levels of disability across the sexes in our cohort (disability was generally worse among men), but it is also possible that the implications of brain structure atrophy may be different in men and women.

### Segmentation and lesion classification

Lesion-TOADS has two major advantages over methods used in previous studies to perform segmentation and classification of brain structures in MS. First, unlike tissue classification tools such as SPM (http://fil.ion.ucl.ac.uk/spm) and FSL's FAST, SIENA, and SIENAX, which only yield CSF, GM, and WM volumes, Lesion-TOADS provides a more detailed subsegmentation for each tissue. Second, neither these methods nor Freesurfer (http://surfer.nmr.mgh.harvard.edu), which is commonly used for brain substructure and cortical segmentation, nor FSL's FIRST (FSL's tool for segmentation of brain substructures), delineates MS lesions as part of the segmentation process. This commonly results in misclassifications, since lesions are often segmented as GM. In all these methods, a separate lesion delineation step, often performed manually or semi-automatically, is required to correct the problem [17], [46]. Lesion-TOADS addresses this issue without any manual interaction.

The results of the analysis with FSL's segmentation tool are different from our results in some important respects. Both approaches confirmed that GM is atrophic in MS, whereas WM does not appear to be. However, the results of the correlation analysis are different. This difference could be as a result of several factors. First, it has been shown recently that even by masking out the lesions prior to SIENAX segmentation, the computed volumes for GM and WM are not accurate, and a more involved correction procedure is required [47]. Second, the segmentation of the subcortical GM structures with the FSL's FAST tissue segmentation tool is not as accurate as its segmentation of cortical GM. Atlas-based approaches such as Lesion-TOADS or FSL's FIRST are necessary for accurate segmentation of subcortical GM. Finally, the difference between the results from the two analyses could also be due to the grouping of all structures in FAST, which can cloud the individual effects we captured in Lesion-TOADS.

### Limitations and future directions

All studies of structure volume alteration in disease states are limited by disease-induced changes in MR properties. This results in signal intensity differences that may bias the tissue segmentation results, which critically depend on tissue-specific differences in signal intensity. Thus, common changes in MS, such as T1 prolongation in GM, WM lesions, and normal-appearing WM, may either accentuate or diminish some of the apparent group differences. Clearly, a more thorough analysis of these effects, which would require extensive simulation and modeling, is necessary.

Lesion-TOADS does not currently segment the globus pallidus. Due to poor contrast between the globus pallidus and adjacent WM on T1-weighted images in both healthy volunteers and MS cases, it is usually included as part of WM in our results. We are currently working on segmenting this structure as part of a future release of the software. Nevertheless, as several studies have reported atrophy of the globus pallidus in MS [12], [44], including the globus pallidus with WM in our analysis cannot explain the lack of WM atrophy.

Additionally, although a cross-sectional analysis of a representative MS cohort, such as the one described here, can offer important insights into the spectrum of disability and brain volume derangements, only a longitudinal study can truly characterize brain structure atrophy and lesion accumulation within individuals [19]. Such a study would also address the confounding factor of brain volume variability even among healthy individuals, a factor that is far from completely controlled by normalizing to the intracranial capacity or including age in the regression analysis. Longitudinal studies are difficult because of issues related to participant retention and accumulation of non-MS pathology over time, and perhaps most importantly variability related to scanner hardware and software changes. However, it is likely that most or all of these problems can be satisfactorily overcome.

Finally, quantitative MR measures such as T1 and T2 relaxation times, magnetization transfer ratio (MTR), and diffusion-derived quantities provide valuable information about disease burden that is not fully captured in brain structure volumes [48]. We are working toward combining these quantitative MR measures with our volumetric analysis in the future.
